# Metastasis of Cancer Stem Cells Developed in the Microenvironment of Hepatocellular Carcinoma

**DOI:** 10.3390/bioengineering6030073

**Published:** 2019-08-23

**Authors:** Said M. Afify, Ghmkin Hassan, Amira Osman, Anna Sanchez Calle, Hend M Nawara, Maram Hussein Zahra, Samah EL-Ghlban, Hager Mansour, Md Jahangir Alam, Hagar A Abu Quora, Juan Du, Akimasa Seno, Yoshiaki Iwasaki, Masaharu Seno

**Affiliations:** 1 Department of Medical Bioengineering, Graduate School of Natural Science and Technology, Okayama University, Okayama 700-8530, Japan; 2 Division of Biochemistry, Faculty of Science, Menoufia University, Shebin El Koum, Menoufia 32511, Egypt; 3 Laboratory of Nano-Biotechnology, Graduate School of Interdisciplinary Science and Engineering in Health Systems, Okayama University, Okayama 700-8530, Japan; 4 Department of Histology, Faculty of Medicine, Kafr Elsheikh University, Kafr Elsheikh 32511, Egypt; 5 Division of Molecular and Cellular Medicine, National Cancer Center Research Institute, Tokyo 104-0045, Japan; 6 Okayama University Research Laboratory of Stem Cell Engineering in Detroit, IBio, Wayne State University, Detroit, MI 48202, USA; 7 Department of Gastroenterology and Hepatology, Graduate School of Medicine, Okayama University, Okayama 700-8558, Japan

**Keywords:** cancer stem cells, metastasis, stem cells

## Abstract

Metastasis develops when cancer cells spread from the primary site of a malignant tumor to the surrounding and distant tissues, and it is the most critical problem in cancer treatment. Our group developed cancer stem cells (CSCs) from induced pluripotent stem cells (iPSCs) in the presence of a conditioned medium (CM) of cancer-derived cells. The CSCs were characterized by the formation of malignant tumors in vivo, followed by metastasis. In this study, CSCs converted from mouse iPSCs in the presence of CM from hepatocellular carcinoma (HCC) cell line Huh7 cells. These converted cells (miPS-Huh7cm cells) were established as the metastatic cells. The generated CSCs were injected into the liver or spleen of nude mice. Almost one month after transplantation, the tumors were excised, and the primary cultured cells derived from the malignant tumors and metastatic nodules were evaluated by stemness and metastatic markers to compare their differences. The miPS-Huh7cm cells exhibited metastatic potential, and efficiently formed malignant tumors with lung and/or liver lesions in vivo, whereas the injected miPS formed teratoma. The primary cultured cells derived from the malignant tumors and metastatic nodules sustained the expression of stemness markers, such as Nanog, Klf4 and c-Myc, and acquired cancer stem markers, such as CD90, CD44 and ALDH1. Simultaneously, the expression of metastatic markers, such as Slug, Twist1 and vimentin, in primary cells derived from the malignant tumors, was higher than in metastatic nodules. The CSCs derived from iPSCs, forming malignant tumors and displaying high metastasis, will provide a good animal model to study the mechanisms of metastasis.

## 1. Introduction

Metastasis is believed to be the cause of more than 90% of cancer mortality, which is an important hallmark of malignant tumors [1,2]. Cancer metastasis is hypothesized to occur through the processes of transient dedifferentiation of epithelial-to-mesenchyme transition (EMT) and the redifferentiation of mesenchyme-to-epithelial transition (MET) of tumor cells as a driving force of metastasis [3,4]. Some reports proved the concept of transient switches of EMT–MET in metastasis [5,6]. 

The tumor cells usually spread to other sites by complex events termed the invasion-metastasis cascade, including several steps, such as invasion, where the tumor cells invade stroma which surround the tumor, intravasation, where the tumor cells enter blood vessels, and finally extravasation, where tumor cells seed in other tissues distant from original sites [2]. In those steps, the EMT is crucial for invasion and metastatic dissemination, and remodeling the extracellular matrix (ECM) by matrix metalloproteinases (MMPs), which are responsible for invasion [7]. In fact, several factors have proved to be critical for inducing EMT; notably Snail, Slug and Twist by downregulation of E-cadherin expression. The metastasis events have been suggested as happening in the late stages of tumor progression. However, many other studies showed that EMT-associated traits and invading events can appear in the early stages in certain preneoplastic lesions [8].

Mesenchyme-to-epithelial transition may contribute to metastasis by driving dissemination of cancer cells to distant locations, where the mesenchymal cells revert to an epithelial phenotype to complete metastasis [9].

On the other hand, several reports have elucidated that tumors are highly heterogeneous, and only a small number of subpopulations within the malignant tumor have the potential to invade the basal membrane and finally metastasize to other organs [10]. Increasing evidence shows the presence of cancer stem cells (CSCs) characterized by self-renewal, differentiation and tumorigenic potential [11]. Although the process of metastasis is still poorly understood, some reports demonstrated that CSCs are responsible for the metastasis. Cancer stem cells associated with metastasis generate a hierarchy of stem-like cells by their differentiation, resulting in the cells initiating metastasis [12]. Some evidence suggested that “metastatic CSCs” could be developed from the original CSCs that evolved throughout tumor progression due to cell plasticity. 

Metastasis models allow the study of how the tumor cells spread from a primary tumor to other organs. Orthotopic transplantation of cancer cells provides an important example of metastasis models, which have the advantage of helping us not only understand the whole metastatic cascade from the initial invasion to metastatic spread through intravasation [13], but also develop new therapies. In this context, intrasplenic injection of CSCs demonstrated in this paper should be one of the most important ways to assess the metastatic potential of tumor cells.

Our group developed a unique CSC model from miPS cells cultured with a cancer cell-conditioned medium that mimicked the conditions of the tumor niche [14]. For this model, our group proposed that CSCs could originate from the normal stem cells/progenitor cells/undifferentiated cells in all tissues chronically affected by the inflammatory microenvironment, which could be defined as the “cancer-inducing niche” [15]. Using this model, we observed that CSCs gave rise to vascular endothelial-like cells by creating a niche that sustained the balance between self-renewal and differentiation and supported the growth of heterogeneous tumors [16]. Furthermore, we proved that CSCs are a key source of Cancer-associated fibroblasts (CAF) cells in the tumor microenvironment [17].

In the present study, we assessed the metastatic potential of CSCs, which were prepared from induced pluripotent stem cells (iPSCs) treated with the conditioned medium of Huh7 cells mimicking the “cancer-inducing niche” to demonstrate that CSCs were responsible for the metastasis from tissue to tissue.

## 2. Materials and Methods 

### 2.1. Cell Culture

miPSCs (iPS-MEF-Ng-20D-17, Lot No. 012) were purchased from Riken Cell Bank (Tokyo, Japan). In the miPSCs, puromycin (puro) resistant genes and *green fluorescent protein* (*GFP*) genes were cloned under the control of *nanog* promoter, so that nanog expression should exhibit puro resistance and green fluorescence in undifferentiated condition. The cells were maintained under a humidified 5% CO_2_ atmosphere at 37 °C on feeder layers of mitomycin-C-treated mouse embryonic fibroblasts (MEFs) (Reprocell, Yokohama, Japan) in miPS medium (Dulbecco’s Modified Eagle Medium (DMEM) containing 15% fetal bovine serum (FBS), 0.1 mM non-essential amino acids (NEAA, Life Technologies), 2 mM L-glutamine, 0.1 mM 2-mercaptoethanol, 1000 U/mL leukemia inhibitory factor (LIF, Millipore, MA, USA) and 50 U/mL penicillin and 50 U/mL streptomycin). Differentiated cells and MEFs were removed by culturing in the presence of 1 μg/mL puro after passaging miPSCs in feeder-less condition. Human HCC cell line Huh7 was obtained from Riken Cell Bank, Japan and maintained in DMEM supplemented with 10% FBS. Then, cells were incubated in a 37 °C incubator with 5% CO_2_. Medium was changed at 80% confluence to 5% FBS. The culture supernatant known as CM was collected after 48 h, centrifuged for 10 min at 1000 rpm at room temperature, and then passed it through sterile 0.22 μm filter (Merck Millipore, MA). The miPS were cultured with CM and miPS medium (1:1) in the absence of LIF and MEF feeder cells; the media were changed every day for 4 weeks. miPS medium containing 15% FBS and LIF used to keep miPSCs surviving and undifferentiated without contact to the CM of Huh7 cells. These cells were used as a control of transplantation.

For primary culture, the tumor derived cells were prepared as follows. The tumors formed by transplantation and metastatic nodules in mice were independently excised and minced into pieces (approximately 1 mm^3^) and washed in Hanks’ Balanced Salt Solution (HBSS) three times. The pieces were suspended and incubated in 2 mL of dissociation buffer, PBS containing 0.25% trypsin, 0.1% collagenase, 20% Knockout™ Serum Replacement (Gibco, NY, USA) and 1 mM of CaCl_2_, at 37 °C for 6 h. Then, 5 mL of DMEM containing 10% FBS was added to terminate the enzyme reaction. The cellular suspension was centrifuged at 300 rpm for 3 min. The supernatant was transferred to a new 15-mL tube then centrifuged at 1000 rpm for 10 min. The cell pellet was resuspended in 5 mL DMEM containing 10% FBS. The cells were cultured in a 60-mm dish coated with gelatin at a density of 3 × 10^5^/dish. Then, the cells were treated with 1 μg/mL puromycin for 1 week to remove the host cells. The expression of GFP and cell morphology was observed and photographed using an Olympus IX81 microscope equipped with a light fluorescence device (Olympus, Tokyo, Japan).

### 2.2. Animal Expermints 

Female 4-week-old Balb/c-nu/nu immunodeficient mice were purchased from Charles River (Kanagawa, Japan). Then, 5 × 10^6^ cells were suspended in sterile HBSS, and intrahepatic and intrasplenic transplantations were performed on immunodeficient mice in a separate group. After 4 weeks, all tumors were resected and sectioned for histologic analysis. All animal experiments were reviewed and approved by the ethics committee for animal experiments of Okayama University under the OKU-2016078.

### 2.3. RNA Extraction and RT-qPCR

Total RNA was extracted from 8 samples using TRIzol RNA isolation reagents (Life Technologies, CA, USA) according to manufacturer’s instructions, and the extracted RNA was treated with DNase I (Promega, Fitchburg, WI, USA) to remove genomic-DNA contamination from samples. RNA concentration was determined by measuring the optical density at 260 nm using a NanoDrop ND-1000 spectrophotometer (Nanodrop Technologies, Wilmington, DE, USA). RNA quality was assessed by combining information from several control steps. Purity was estimated from the absorption ratios using the NanoDrop. Only the RNA samples with A260/A280 ratio in the range between 1.8 and 2.0 were used for further experiments.

Four micrograms of RNA were used to synthesize cDNA using a GoScript™ Reverse Transcription System kit (Promega, Fitchburg, WI, USA) following the kit’s instructions. The resultant cDNA was diluted to 1:10 in nuclease-free water and stored in aliquots at −80 °C until use. Then, RT-qPCR was conducted using a Light Cycler 480 SYBR green I Master Mix (Roche Diagnostics GmbH, Mannheim, Germany), following the manufacturer’s instructions, and melting curve analysis was also done to confirm the specificity of amplification. All primers were designed by bioinformatics tool Primer-BLAST at NCBI (https://www.ncbi.nlm.nih.gov/tools/primer-blast/index.cgi?LINK_LOC=BlastHome) to ensure primer specificity. Primers used in this study are listed in Table 1. Different samples from different passages were analyzed to assess the stability of GAPDH, β-actin and β-tubulin as housekeeping genes. Resultantly, GAPDH was found the most stable and used as reference gene. Data were analyzed by LightCycler® 480 Software (Roche Diagnostics GmbH, Mannheim, Germany) to calculate Cp values. All the data were normalized by GAPDH expression following the manufacturer’s instructions.

Primers used are listed in Table 1.

### 2.4. Flow Cytometry 

Flow cytometry analysis was conducted on cultured cells, where around (5 × 10^5^) cells were suspended in PBS and GFP expression was detected by BD AccuriTM C6 Plus (BD, USA). Data were analyzed with Flowjo software (Treestar Inc., San Carlos, CA, USA).

### 2.5. Histological Analysis

#### 2.5.1. Hematoxylin and Eosin Staining 

Sections were stained with Hematoxylin 0.5% (Sigma-Aldrich, St. Louis, MO, USA) and Eosin Y (Sigma-Aldrich, St. Louis, MO, USA). Slides were analyzed using a microscope (Eclipse Ti, Tokyo, Japan).

#### 2.5.2. Immunohistochemistry 

Immunohistochemistry (IHC) was performed the same as standard procedures. Briefly, 5 mm tissue sections were deparaffinized in xylene and rehydrated through a gradual decrease in the concentration of ethanol. The antigen epitopes were then unmasked using sodium citrate buffer (pH 6.0) by a standard microwave heating technique. After hydrogen peroxide blocking and normal serum blocking, Ig Blocking Reagent (Vector Laboratories, Burlingame, CA, USA) was used as a blocking buffer. Subsequently, the sections were incubated overnight at 4 °C with the following primary antibodies: CD44 (abcam, ab24504), GFP (cell signaling, #2956), Ki67 (abcam, ab66155), Snail/Slug (abcam, ab180714), CK19 (abcam, ab15463), E-Cadherin (cell signaling, #3195), Vimentin (abcam, ab45939) and N-cadherin (abcam, ab18203). After primary incubation, sections were incubated with the appropriate biotinylated secondary antibodies (Vector Laboratories, Burlingame, CA, USA), followed by incubation with the ABC reagent (Vector Laboratories, Burlingame, CA, USA). Detection was accomplished using DAB (3,3’-diaminobenzidine) substrates (Vector Laboratories, Burlingame, CA, USA). Incubation of sections with phosphate-buffered saline (PBS) served as negative controls. Sections were lightly counter-stained with hematoxylin and mounted. The IHC-stained slides were mounted in Micromount (Leica, Nussloch, Germany).

### 2.6. Statistical Analysis

Data from three independent experiments and mean values were presented as mean ± SD at least three times determinations and analyzed by Student’s *t*-test, as well as one-way analysis of variance (ANOVA). A *p*-value less than 0.05 was considered to be statistically significant.

## 3. Results

### 3.1. Intrahepatic Transplantation

#### 3.1.1. miPS-Huh7cm Cells Metastasized to the Lung

**Figure 1:**
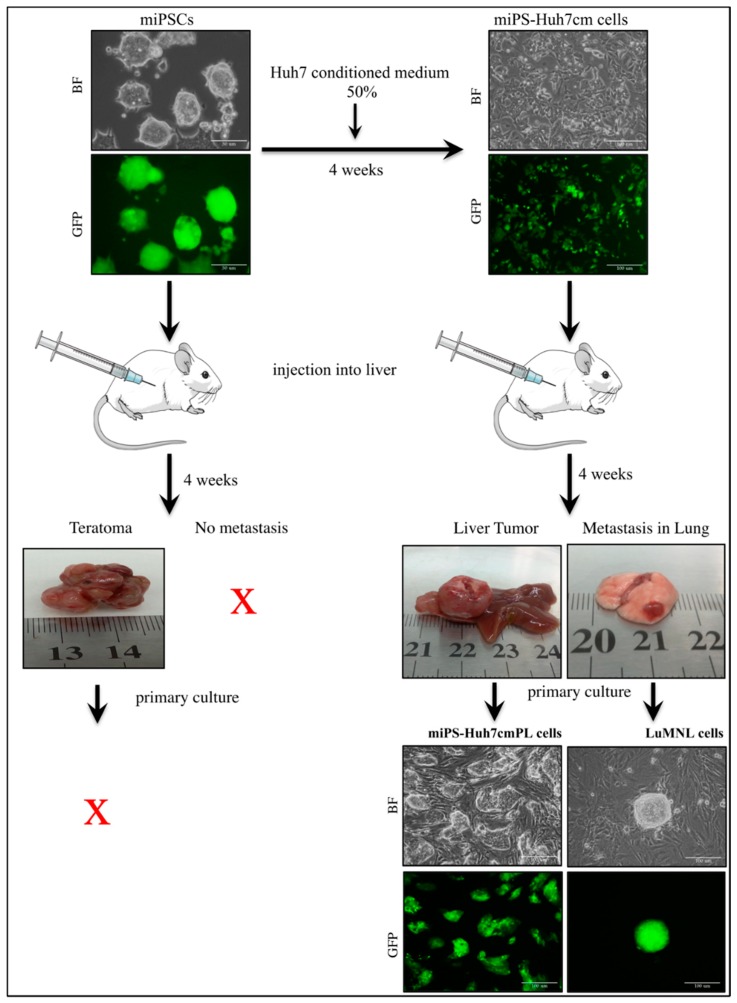
This representative image shows the conversion of mouse induced pluripotent stem cells (miPSCs) into cancer stem cells (CSCs) in the presence of Huh7 cells conditioned medium (CM) and shows the appearance of an orthotopic malignant tumor in the liver, with lung metastasis from miPS-Huh7cm cells, whereas miPSCs formed teratomas after 4 weeks. Scale bars represent 50 μm. The primary cultures from the malignant tumor and lung metastasis showed green fluorescent protein (GFP)-expressing cells indicating the injected cells, whereas primary cells from teratoma cannot survive. Scale bars represent 100 μm.

We have reported that CSCs could be induced from miPSCs in the presence of a cancer-inducing niche derived from cancer cell lines without genetic manipulations, as reviewed by Afify and Seno [11]. The CSCs have been characterized in different ways to analyze the mechanisms of CSC induction in vitro and in vivo. In this study, miPSCs were treated with the CM derived from hepatocellular carcinoma cell line Huh7 cells, to convert miPSCs into CSCs in the absence of LIF. After 4 weeks of treatment, the converted cells showed high metastatic potential in vitro compared to miPSCs which was confirmed using Matrigel invasion assay (scale bars represent 100 μm, Supplementary Material). Then, the converted cells were implanted in the liver of mice to check the metastatic potential in vivo. Almost one month later, a malignant tumor developed in the liver together with metastatic nodules in the lung while teratoma developed from untreated miPSCs without metastasis (Figure 1). 

The primary cultured cells isolated from liver tumor were named miPS-Huh7cm PL cells, and those from lung metastatic nodule cells were named LuMNL cells. As both primary cultured cells were positive for GFP signal when they made spheres, they were confirmed to be derived from miPS-Huh7cm cells and not from host tissues, whereas the cells derived from teratoma could not survive for more than one week.

**Figure 2:**
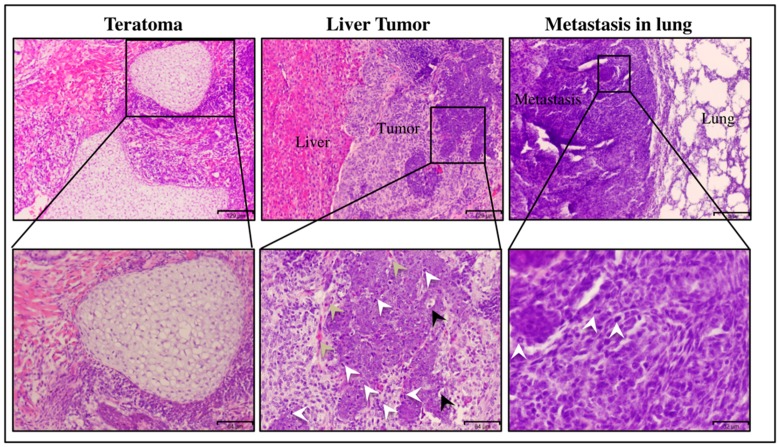
Histopathological analysis of representative hematoxylin–eosin-stained sections of malignant tumor in the liver (scale bars represent 129 μm), which showed abnormal mitotic figures (white arrow), cellular infiltration of blood vessels (bale yellow arrow), cytoplasmic degeneration (black arrow) and high nuclear-to-cytoplasmic ratio. Scale bars represent 64 μm. The lung metastatic nodules show the mitotic figures (white arrow), (scale bars represent 32 μm) and tumor derived from miPS, which showed teratoma phenotype (scale bars represent 129 μm).

The primary tumor in the liver exhibited a poorly differentiated phenotype, high nuclear-to-cytoplasmic ratio, cytoplasmic degeneration and abnormal mitotic figures, which were signs of malignancy (Figure 2). The metastatic nodules in lung also showed high mitotic figures. This observation is consistent with extra-hepatic metastasis, which is most often found as lung metastasis in the case of liver cancer. As the control, miPSCs tumors had a teratoma-like phenotype.

**Figure 3:**
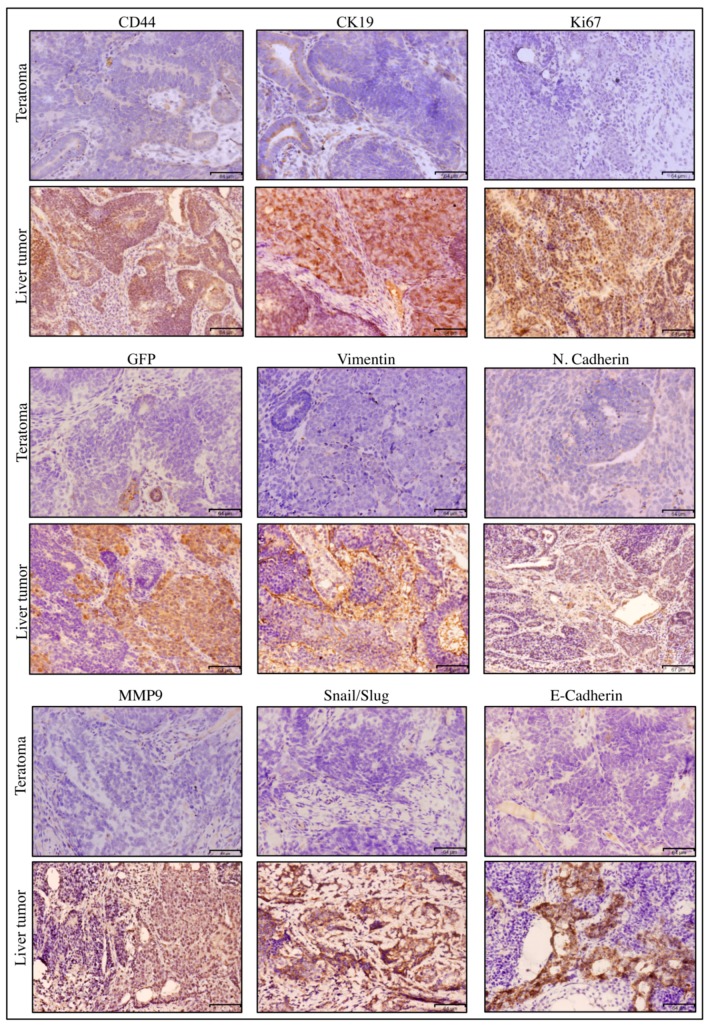
Immunohistochemistry (IHC) of miPS-Huh7cm cell-derived primary tumor in liver against CD44, CK19, Ki67, GFP, Vimentin, N-cadherin, MMP9, Snail/Slug and E-cadherin compared to miPSC-derived teratoma. Scale bars represent 64 μm.

The tumor tissue derived from miPS-Huh7cm cells was found to be rich in CSCs expressing CD44, liver CSC marker, CK19 and interphase marker Ki67, as well as GFP, indicating the presence of miPSHuh7cm-derived cells (Figure 3). Vimentin and N-cadherin implied the presence of mesenchymal cells, which should be responsible for metastasis. Also, MMP9 and Snail/Slug were detected in the tumor tissue, suggesting the metastatic potential of the tumor. Together with Vimentin and N-cadherin, E-cadherin was found, indicating that the tumor tissue is composed of a heterogeneous population of epithelial and mesenchymal cells. From those observations, it could be concluded that miPS-Huh7cm cells were CSCs with tumorigenic and metastatic potential in vivo.

#### 3.1.2. Characterization of miPS-Huh7cm Cells, Primary Tumor Derived Cells and Lung Metastatic Cells 

**Figure 4:**
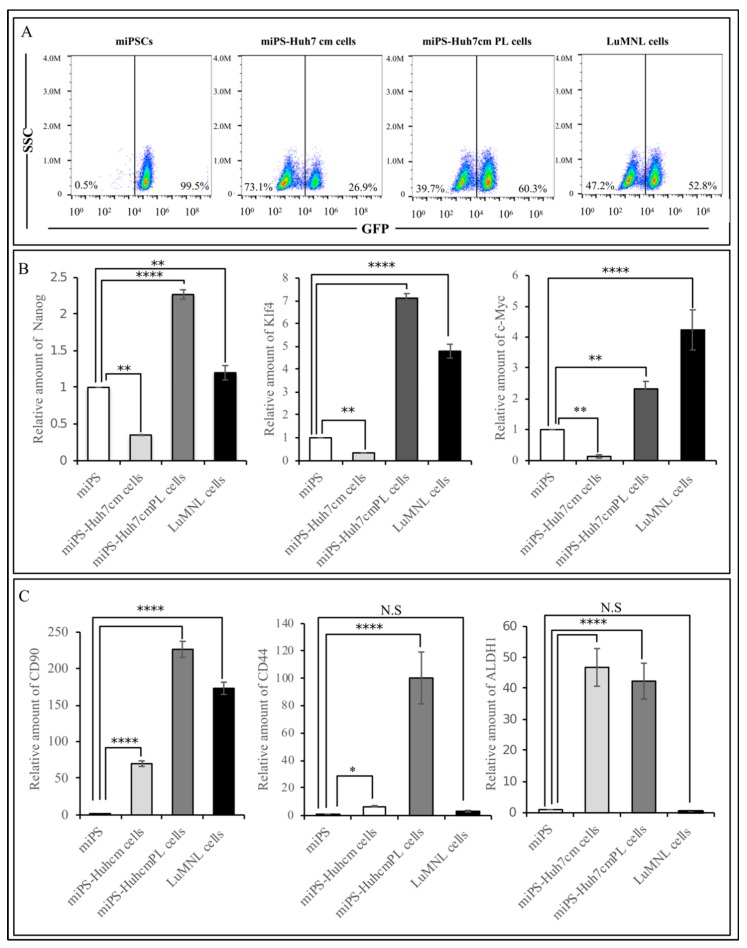
(**A**) FACS analysis shows green fluorescent protein (GFP) population in miPS-Huh7cm, miPS-Huh7cm PL cells and LuMNL cells; (**B**) RT-qPCR analysis of stemness markers in miPS-Huh7cm, miPS-Huh7cm PL cells and LuMNL cells in, comparison with miPSCs; (**C**) RT-qPCR analysis of CSC markers in miPS-Huh7cm and miPS-Huh7cm PL cells and LuMNL cells in comparison with miPSCs (* *p* < 0.01, ** *p* < 0.001, *** *p* < 0.0001, **** *p* < 0.00001).

In an adhesive culture, both miPS-Huh7cmPL and LuMNL cells exhibited two different types of populations; one was a colony expressing GFP and the other was fibroblast-like cells attached to the bottom of the dish without expressing GFP (Figure 1). The ratio of GFP positive and negative cells was estimated by flow cytometry (Figure 4A). The three cells contained GFP positive cells between 20% and 65%, whereas undifferentiated miPSCs were all GFP positive. 

By RT-qPCR analysis, miPS-Huh7cmPL cells and LuMNL cells were confirmed to sustain the expression of endogenous stemness markers, Nanog, Klk4 and c-Myc, as much as miPSCs and miPS-Huh7cm cells (Figure 4B). On the other hand, the expression of CSC-markers such as CD90, CD44 and ALDH1 was confirmed in miPS-Huh7cm cells, whereas the expression of those markers was significantly elevated in miPS-Huh7cmPL cells when compared to those in miPS-Huh7cm cells, LuMN cells and miPSCs (Figure 4C). 

**Figure 5:**
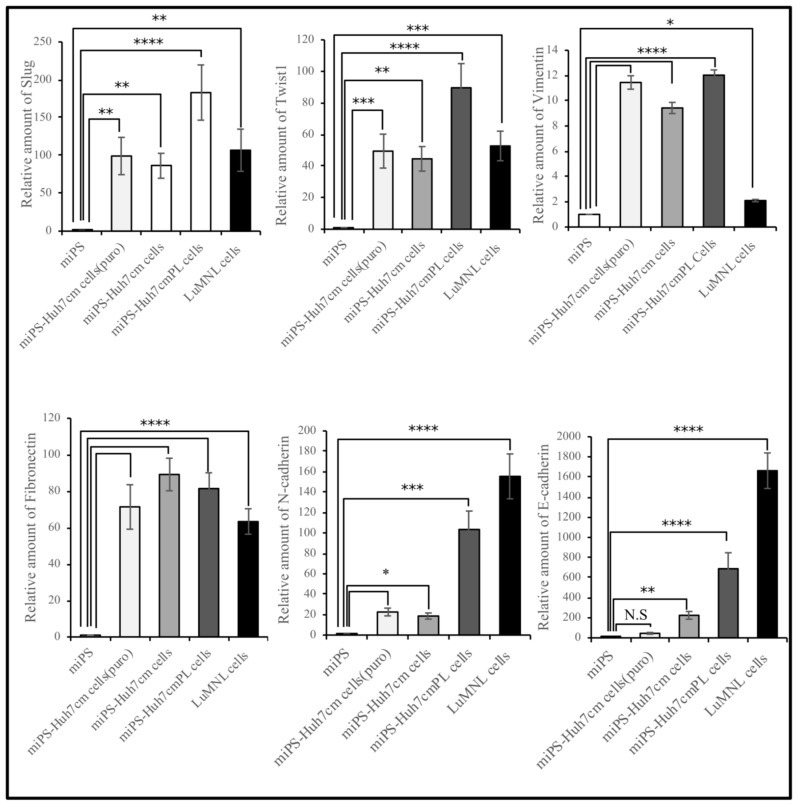
Real-time-qPCR analysis of metastatic markers (Slug and Twist1), mesenchymal markers (Vimentin, N-cadherin and fibronectin) and the epithelial marker (E-cadherin) in cells derived from intrahepatic transplantation (miPS-Huh7cmPL cells and LuMNL cells) in comparison with miPS-Huh7cm cells, miPS-Huh7cm cells (Puro) and miPSCs (* *p* < 0.01, ** *p* < 0.001, *** *p* < 0.0001, **** *p* < 0.00001).

The expression of metastatic markers, such as Slug, Twist1 and Vimentin was significantly different (*p* < 0.01) between miPS-Huh7cmPL and LuMNL cells, whereas fibronectin expressed an equivalent level (Figure 5). The miPS showed little or very low expression of metastatic markers, which is inconsistent with the result that miPSCs showed teratoma formation without metastasis (Figure 1). The N-Cadherin expression was elevated more than 100-fold in both miPS-Huh7cmPL and LuMNL cells, whereas it was elevated 10- to 20-fold in miPS-Huh7cm when compared to miPSCs. 

Furthermore, LuMNL cells showed significantly higher expression of E-Cadherin, more than double when compared to miPS-Huh7cmPL cells (*p* < 0.001). Collectively, all these data suggest that miPS-Huh7cm cells should undergo MET before the cells at the site of metastasis partly differentiate into an epithelial phenotype. 

### 3.2. IntraSplenic Transplantation 

#### 3.2.1. miPS-Huh7cm Cells Metastasized to Liver and Lung

**Figure 6:**
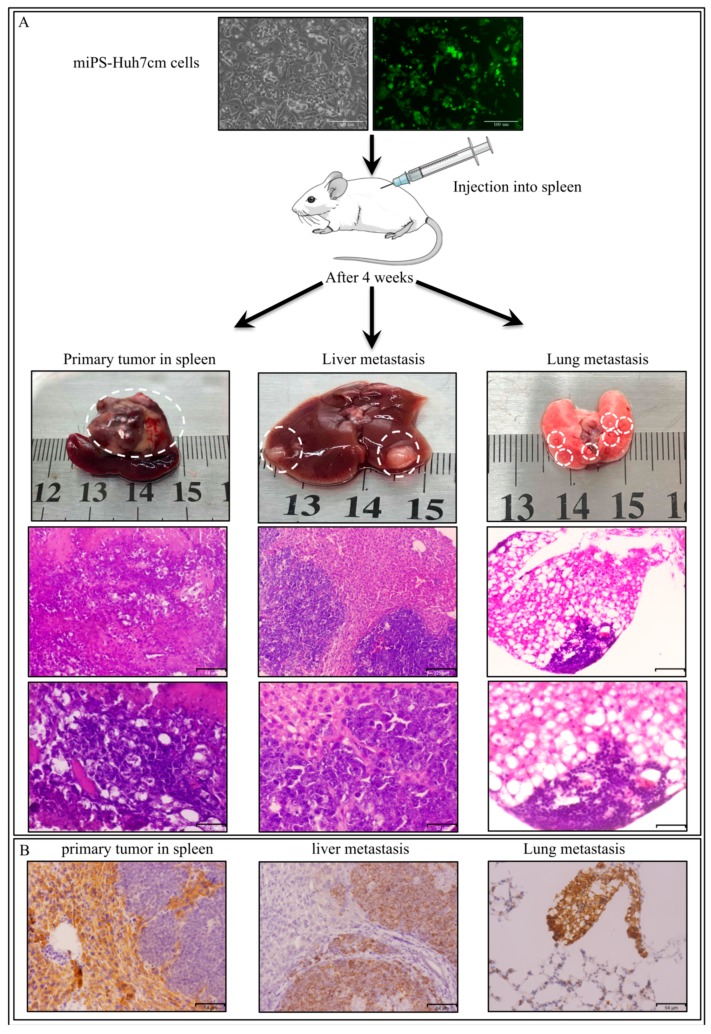
(**A**) Representative scheme of intrasplenic transplantation of miPS-Huh7cm cells and histological examination of derived tumors in different tissues. The results showed tumor formation in spleen, a liver metastasized tumor and lung-metastatic nodules. Scale bars represent 129 μm and 32 μm. (**B**) Immunohistochemistry of derived tumors against GFP which confirmed that miPS-Huh7cm cells were metastatic cells. Scale bars represent 64 μm.

Furthermore, to investigate the metastatic potential of miPS-Huh7cm cells, intrasplenic transplantation was performed. Balb/c-nu/nu mice intrasplenically received 0.5 × 10^5^ miPS-Huh7cm cells. After 30 days from intrasplenic transplantation, three out of three mice presented splenic tumors with liver metastasis, as well as distant metastases in the lung (Figure 6A). 

The primary tumor in spleen showed a malignant phenotype of high nuclear-to-cytoplasmic ratio, and high mitotic figures (Figure 6A). Also, liver metastatic tumor or lung metastatic nodules showed a poorly differentiated phenotype with high mitotic figures. This observation indicates the metastatic potential of miPS-Huh7cm cells. Furthermore, IHC staining of GFP confirms that the metastatic cells were the undifferentiated miPS-Huh7 cells, but neither differentiated cancer cells, nor the cells derived from the host (Figure 6B).

#### 3.2.2. Characterization of Primary Cells Derived from Spleen Tumor, Liver Metastatic Tumor and Lung Metastatic Nodules

**Figure 7:**
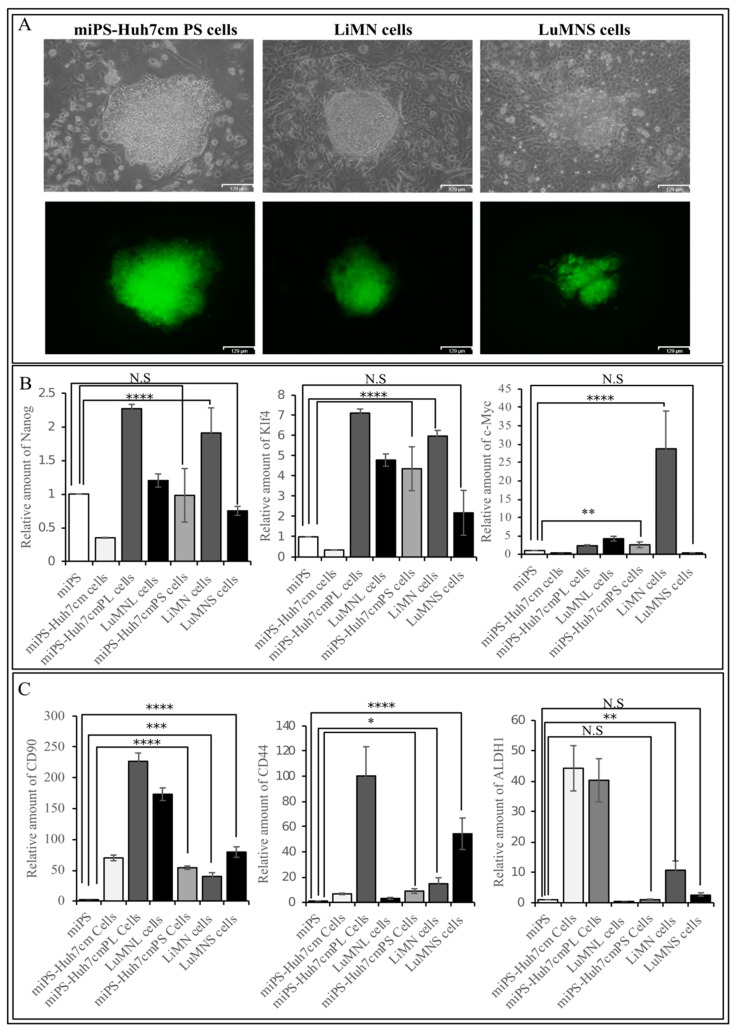
(**A**) Representative images for adherent cultures of miPS-Huh7cmPS cells, LiMN cells and LuMNS cells. Images show the differentiated cells and undifferentiated GFP-positive cells. Scale bars represent 129 μm; (**B**) RT-qPCR analysis of CSC markers (CD44,CD90,ALDH1) in cells derived from intrahepatic transplantation (miPS-Huh7cmPL cells and LuMNL cells) and intrasplenic transplantation-derived cells (miPS-Huh7cmPS cells, LiMN cells and LuMNS cells) in comparison with miPS-Huh7cm cells and miPS; (**C**) RT-qPCR analysis of stemness markers (Nanog, Klf4 and c-Myc) in cells derived from intrahepatic transplantation (miPS-Huh7cmPL cells and LuMNL cells) and intrasplenic transplantation-derived cells (miPS-Huh7cmPS cells, LiMN cells and LuMNS cells) in comparison with miPS-Huh7cm cells and miPS (* *p* < 0.01, ** *p* < 0.001, *** *p* < 0.0001, **** *p* < 0.00001).

The primary culture derived from the primary tumor in spleen was named miPS-Huh7cmPS; the cells derived from metastatic liver tumor were named LiMN cells and the cells derived from metastatic lung nodules were named LuMNS cells. The three types of cells were positive for GFP when they formed spheres, indicating that the cells were derived from miPS-Huh7cm cells and not from the host (Figure 7A).

The miPS-Huh7cmPS cells, LiMN cells and LuMNS cells were confirmed to express the endogenous stemness markers, such as Nanog, Klf4 and c-Myc, at a comparable level to miPSCs, and the cells derived from intrahepatic transplantation. As for the expression of endogenous Nanog, the three types of cells derived from intrasplenic transplantation showed lower expression when compared to miPSCs or miPS-Huh7cm cells, but no significant difference when compared to cells derived from intrahepatic transplantation (Figure 7B). The three types of cells derived from intrasplenic transplantation showed the elevation of Klf4 expression in the same manner as the cells from intrahepatic transplantation when compared to miPSCs. The LiMN cells showed a significantly higher expression of c-Myc when compared to all other cells.

To evaluate the difference between metastasis derived from intrahepatic and intrasplenic injection, and the property effect of changing the site of injection on metastatic and CSCs markers, cells from both intrahepatic and intrasplenic transplantation were compared to each other using qPCR analysis. All cells showed an elevation of CSC marker expression, such as CD90, CD44 and ALDH1, when compared to miPSCs (Figure 7C). A high magnitude of enhancement in the expression of the CSC markers was observed in miPS-Huh7cmPL cells.

All cells sustained the expression of stemness markers, such as Nanog, Kif4 and c-Myc. The expression of those markers in LuMNL cells was higher than those in LuMNS cells. The LuMNL cells showed a tremendously high expression of CD90, whereas expression of both CD44 and ALDH1 was lower when compared to LuMNS cells.

**Figure 8:**
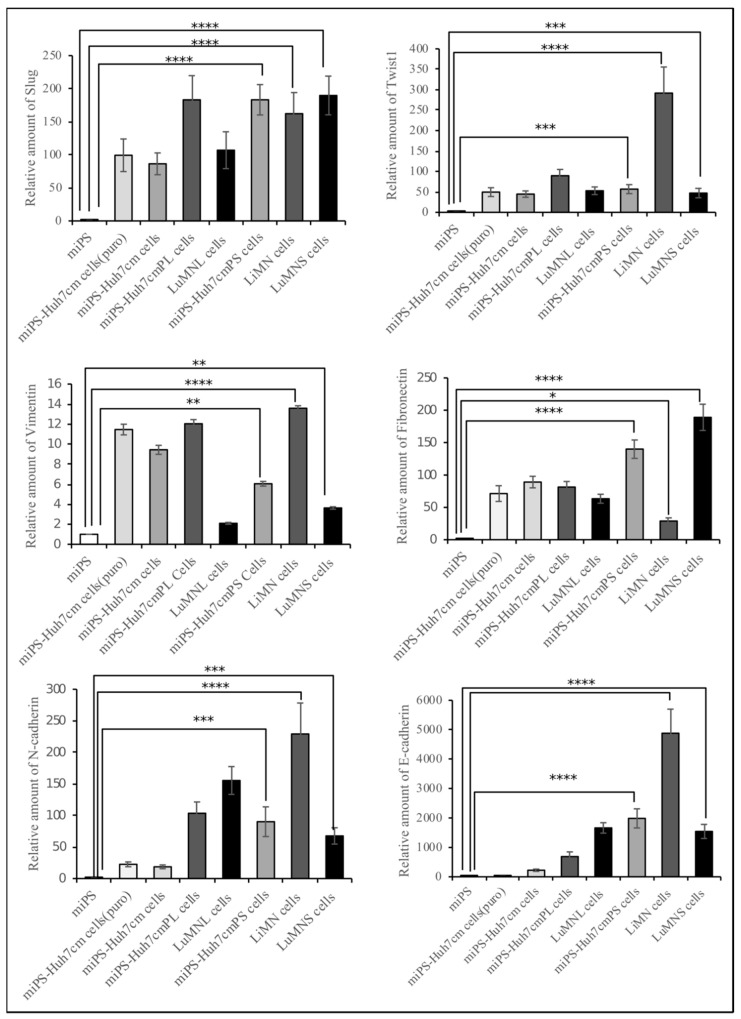
Real-time-qPCR analysis of metastatic markers (Slug and Twist1), mesenchymal markers (Vimentin, N-cadherin and fibronectin) and epithelial marker (E-cadherin) in cells derived from intrahepatic transplantation (miPS-Huh7cmPL cells and LuMNL cells) and intrasplenic transplantation-derived cells (miPS-Huh7cmPS cells, LiMN cells and LuMNS cells) in comparison with miPS-Huh7cm cells and miPS (* *p* < 0.01, ** *p* < 0.001, *** *p* < 0.0001, **** *p* < 0.00001).

Cells derived from both intrahepatic and intrasplenic transplantation showed equivalently high expression of metastatic markers Slug, Twist1, N-cadherin, Vimentin, fibronectin and E-cadherin when compared to miPSCs (Figure 8). The comparison of primary cultured cells, miPS-Huh7cmPL cells and miPS-Huh7cmPS cells, showed no significant difference between the expression of Slug and N-Cadherin. The miPS-Huh7cmPL cells showed a relatively higher expression of Twist1 and Vimentin than miPS-Huh7cmPS cells, whereas miPs-Huh7cmPS cells showed a relatively higher expression of E-cadherin than miPS-Huh7cmPL cells. Significant differences in the expression of Slug and fibronectin were found between LuMNS cells and LuMNL cells, even though both were from lung metastasis. In contrast, LiMN cells showed the highest expression of Twist1, Vimentin, N-cadherin and E-cadherin among all cells.

**Figure 9:**
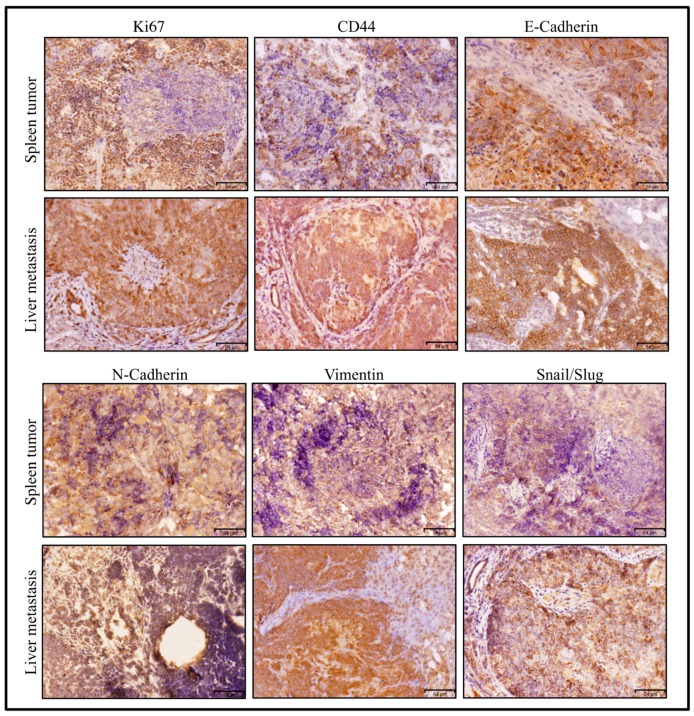
Immunohistochemistry of a miPS-Huh7cm cell-derived tumour in the spleen and liver—a metastatically developed tumor via intrasplenic injection, which is shown in Figure 6. The tissue section was stained with antibodies against CD44, Ki67, GFP, Vimentin, N-cadherin E-cadherin and Snail/Slug. Scale bars represent 64 μm.

According to the results of RT-qPCR (Figure 8), the metastatic tumor in liver was assessed for several markers compared to the primary tumor in spleen (Figure 9). Strong immunoreactivity to metastatic marker, Slug/Snail, mesenchymal markers, Vimentin and N-cadherin, epithelial marker, E-cadherin, and proliferative marker Ki67 was found in immunohistochemical observation of the metastatic tumor in liver compared to the primary tumor in spleen. This observation indicates the presence of a subpopulation of CSCs, which was confirmed by CD44. 

## 4. Discussion

Metastasis is one of the most severe events in cancer. The metastatic process depends not only on the characteristics of the primary tumor cells but also on the tumor microenvironments, presence of immune cells, extracellular matrix and stromal barriers [18,19,20]. Metastasis also depends on the target tissue, which will provide the microenvironments, allowing the settlement of the metastatic cells and the growth of metastatic tumor. To develop a novel and effective therapy, the molecular events that drive the metastasis following the progression of primary cancer should be understood more precisely.

Many reports described the link between CSCs and the metastatic potential of the malignant tumor [21,22,23]. They suggested that CSCs generated cellular heterogeneity by installing a diverse hierarchy leading to a range of distinct cell types present within the tumor [24]. This heterogeneity is considered responsible for metastasis [25].

Our group previously established a protocol to generate CSCs by culturing miPSCs in the CM from cancer cell lines. Through the experiments, the CM from cancer-derived cells was suggested to be rich in the secreted factors that potentially mimic the tumor microenvironment [14,15,16,17,26,27,28]. In this study, a model of metastasis to the liver and lung was established by orthotopic transplantation of CSCs derived from miPSCs treated with the CM of Huh7 cells. 

The CM from Huh7 cells successfully converted miPSCs into CSCs, which developed a malignant tumor in the liver 4 weeks after intrahepatic transplantation (Figure 1). This malignant tumor metastasized to the lung, which was considered to be the most common target tissue in extrahepatic metastasis [29].

Cells derived from intrahepatic transplantation miPS-Huh7cm PL cells showed high expression of CSC markers, such as CD90, CD44 and ALDh1, while LuMNL cells showed decreases in the three CSC markers, CD90, CD44 and ALDH1. The observed decrease was high in CD44 and ALDH1 but small in CD90. These results of decreased expression might be explained by the differentiation undergone by MET when the CSCs metastasized to lung from liver. Many reports take the expression of CD90 for granted, as a marker of liver CSC, as well as mesenchymal cells [30,31]. Therefore, we could demonstrate that CD90 was highly expressed in liver. The expression of CD90 in LuMNL cells indicated the cells was originated from the liver tumor. Furthermore, E-cadherin showed high expression as same as N-cadherin in miPS-Huh7cmPL and LuMNL cells. These results agree with the demonstration by other group that not all the mesenchymal markers, such as Vimentin, N-cadherin and fibronectin, change the expression level in a mode of epithelial marker opposite to E-cadherin. Those scientists reported that positive expression of both E-cadherin and N-cadherin in more than 50% of cases showed a poor survival rate compared to cancer with only one positive marker [32]. The high-level expression of is probably because of the presence of many different subpopulations localized in the same tumor tissue, which reflect the heterogenicity of tumor tissue. In this context, LuMNL cells showed significantly higher expression of E-Cadherin, more than double of that in miPS-Huh7cmPL cells due to MET. Accordingly, the expression levels of E-cadherin and N-cadherin in miPS-Huh7cmPL cells were higher than those in miPS-Huh7cm cells. These sequential upregulations could be explained by the differentiation protentional of CSCs caused by MET.

On the other hand, intrasplenic transplantation of tumor cells is a well-known effective method to develop liver metastases [33]. The miPS-Huh7cm cells exhibited liver metastasis, which was followed by lung metastasis, with 100% efficiency in immunodeficient mice after 4 weeks of intrasplenic transplantation. These results agree with other groups in demonstrating that intra-splenic injection model displayed the fastest approach to develop a liver metastasis mouse model [34,35]. The main advantage of our model, is the lower number of injected cells, as in our study only 0.5 × 10^6^ cells were injected, while the other study required 2 × 10^6^ cells to achieve metastasis. Additionally, the development of lung metastatic nodules in our study was considered another advantage, that indicated the possibility of re-metastasis from liver to lung and the opportunity for testing therapeutic agents targeting metastasis in the liver and lung.

Only one tumor formed in the spleen, whereas we observed multiple metastatic nodules in different liver lobes and numerous small nodules in the lung. From the sizes of the tumor and nodules, the metastasis appears to occur sequentially from spleen to liver and then to the lung as a result of re-metastasis of the metastatic tumor that grew in the liver, a process described by Rashidi et al. [36].

Although the primary culture of the tumors developed from both methods of transplantation, showing the expression of CSC and stemness markers, the intrasplenic transplantation-derived cells showed lower expression of stemness and CSC markers when compared to intrahepatic transplantation-derived cells, probably due to the tumor microenvironment [37].

Transplantation and metastasis exhibited a different signature of tumor development even in the same tissue. The pattern of gene expression related to stemness, CSC markers, metastatic markers and mesenchymal markers between the primary liver tumor from intrahepatic transplantation and LiMN cells was completely different in vitro (Figure 7 and Figure 8) and in vivo (Figure 3 and Figure 9). 

It is worth noting that LiMN cells showed the highest expression in c-Myc, which was related to metastatic potential and correlated to metastatic markers, when compared to all primary cells derived from intrahepatic and/or intrasplenic transplantation. This finding is consistent with other reports, which demonstrated that high c-Myc expression is detected in metastatic hepatic tumors compared with primary liver tumor [38]. Despite transplantation in different places, liver and spleen, the metastasized tumor that developed in the lung exhibited similar signatures.

## 5. Conclusions

The metastatic potential of CSCs developed from miPSCs was evaluated by transplantation in the liver and spleen. These models exhibited the upregulation of different sets of genes related to metastasis and stemness. This approach of the transplantation of CSCs should be proposed as a suitable model for therapeutic studies of metastasis. 

## Figures and Tables

**Table 1:** Primers used in RT-qPCR reactions.

Gene	NM	Forward Primer	Reverse Primer
**GAPDH**	NM_008084	AACGGCACAGTCAAGGCCGA	ACCCTTTTGGCTCCACCCTT
**Nanog**	NM_028016.3	AGGGTCTGCTACTGAGATGCTCTG	CAACCACTGGTTTTTCTGCCACCG
**Klf4**	NM_010637.3	GGACTTACAAAATGCCAAGGGGTG	TCGCTTCCTCTTCCTCCGACACA
**CD44**	NM_009851.2	AGAAAAATGGCCGCTACAGTATC	TGCATGTTTCAAAACCCTTGC
**CD90**	NM_009382.3	TGCAGCTAGGGGAGTCCAGAAT	TCCAGGCGAAGGTTTTGGTT
**ALDH1**	NM_001361503.1	AACACAGGTTGGCAAGTTAATCA	TGCGACACAACATTGGCCTT
**Slug**	NM_011415.3	CACACACACAGTTATTATTTCCCCA	ACTTACACGCCCCAAGGATG
**Twist1**	NM_011658.2	GCCGGAGACCTAGATGTCATTGT	TTAAAAGTGTGCCCCACGCC
**E-cadherin**	NM_009864.3	AACCCAAGCACGTATCAGGG	GGGGTCTGTGACAACAACGA
**N-cadherin**	NM_007664.5	CCTTGCTTCAGGCGTCTGTG	CTTGAAATCTGCTGGCTCGC
**Vimentin**	NM_011701.4	GCCCTTAAAGGCACTAACGAG	ATTCACGAAGGTGACGAGCC
**Fibronectin**	NM_010233.2	CCAACCTCTTGGTGCGCTA	AATCGAGACCTGTTTTCTGCCT

## Supplementary Materials

The following are available online at https://www.mdpi.com/2306-5354/6/3/73/s1.

## Abbreviations

CSC	cancer stem cell
iPSCs	induced pluripotent stems
EMT	epithelial-to-mesenchyme transition
MET	mesenchyme-to-epithelial transition
LIF	leukemia inhibitory factor
miPS-Huh7cmPL cells	primary cultured cells isolated from liver tumor
LuMNL cells	lung metastatic nodule cells in case of intrahepatic transplantation
miPS-Huh7cmPS cells	primary culture derived from tumor in spleen
LiMN cells	cells derived from metastatic liver tumor
LuMNS cells	cells derived from metastatic lung nodules
GFP	green fluorescent protein
MEFs	mouse embryonic fibroblasts
MMP	matrix metalloproteinase
