# Supplementary figures and images for: Metastasis of Cancer Stem Cells Developed in the Microenvironment of Hepatocellular Carcinoma
Source: Bioengineering (Basel). 2019 Aug 23;6(3):73. doi: 10.3390/bioengineering6030073 (PMC6784246; doi:10.3390/bioengineering6030073)

A

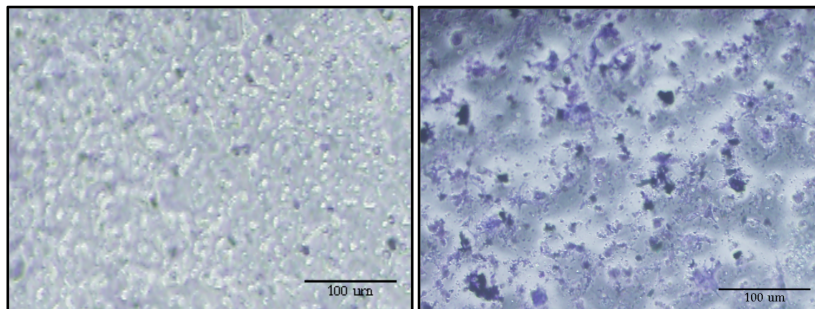

B

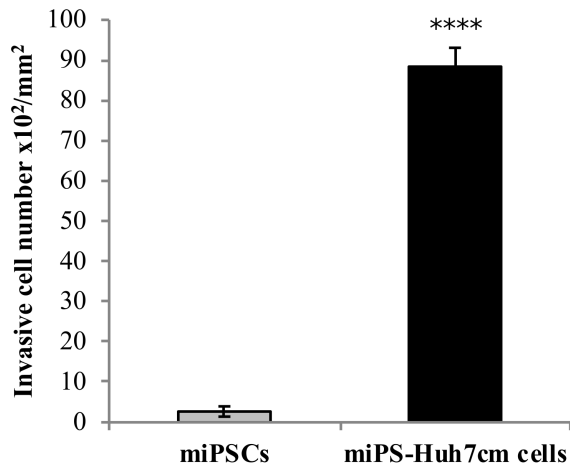

Supplement: Supplementary file 1 [file bioengineering-06-00073-s001.pdf]
